# Case–control study on risk factors for in-hospital mortality in patients with severe COVID-19

**DOI:** 10.3389/fpubh.2024.1424720

**Published:** 2024-10-08

**Authors:** Kemei Wu, Lili Yin, Jiangqin Han, Qiuhan Cai, Yang Guo, Xin Jin, Jinling Wu, Yupei Cheng

**Affiliations:** ^1^Graduate School, Tianjin University of Traditional Chinese Medicine, Tianjin, China; ^2^National Clinical Research Center for Chinese Medicine Acupuncture and Moxibustion, First Teaching Hospital of Tianjin University of Traditional Chinese Medicine, Tianjin, China

**Keywords:** case–control study, mortality, retrospective studies, risk factors, risk prediction models, severe COVID-19

## Abstract

**Objective:**

The purpose of this study was to identify independent risk factors affecting patient survival and explore predictors of severe cases of coronavirus disease 2019 (COVID-19).

**Methods:**

We conducted a retrospective, observational, case–control study on adult patients with severe COVID-19 who were admitted to affiliated hospitals in Tianjin between December 18, 2022, and January 31, 2023. We used univariate and multifactorial logistic regression analyses to analyze demographic indicators, comorbidity profiles, and laboratory parameters in two groups of patients (deceased and surviving) to identify independent risk factors for death in patients with severe COVID-19.

**Results:**

Patients in the deceased group were older than those in the survival group (*p* = 0.018), and there were more cases of coexisting respiratory insufficiency in the deceased group (*p* = 0.002). Additionally, laboratory test results for white blood cell count (WBC) and creatine kinase (CK) showed significantly higher values in the deceased group (*p* = 0.047 and *p* = 0.029, respectively), while arterial oxygen partial pressure (PAO2) showed significantly lower values compared to the survival group (*p* = 0.021). Age, respiratory insufficiency, WBC_H_ (highest WBC value), CK_H_ (highest CK value), and PAO2_F_ (first PAO2 value) had area under curve (AUC) values of 0.698, 0.838, 0.721, 0.744, and 0.633, respectively.

**Conclusion:**

The main risk factors for mortality in patients with severe COVID-19 that we identified in this study were the advanced age of patients, coexisting respiratory insufficiency, elevated levels of WBC and CK, and decreased levels of PAO2. Elevated WBC and CK laboratory parameters, in particular, demonstrated good predictive value for in-hospital mortality risk.

## 1 Introduction

COVID-19, caused by the severe acute respiratory syndrome coronavirus type 2 (SARS-CoV-2) (1), has emerged as a significant public health crisis warranting international concern (2). This viral outbreak poses a serious threat to global health security with its substantial morbidity and mortality (3). As of August 2024, the global cumulative number of reported COVID-19 cases has exceeded 700 million, with over 7 million deaths. Although COVID-19 has eased globally, some countries continue to be severely affected. China successfully contained the virus’s spread through strict control measures in the early stages of the pandemic (4–6), but later, the emergence of variants and adjustments in control strategies led to increases in cases and deaths. As of August 2024, China’s cumulative death toll is approaching 120,000. India is one of the countries most severely impacted by the COVID-19 pandemic. Since the start of COVID-19, India has reported over 44 million confirmed cases and nearly 520,000 deaths. In mid-2021, widespread transmission of the Delta variant led to a rapid worsening of the situation in India, putting immense pressure on hospitals and medical resources, and significantly increasing the number of deaths during the peak of the pandemic. Brazil’s COVID-19 situation is similarly severe, with over 36 million confirmed cases and close to 700,000 deaths. Brazil’s case and death numbers peaked in 2021 and 2022. Despite its smaller population, Ecuador was also heavily impacted by the pandemic. Particularly in the early stages, the situation in Ecuador was extremely severe due to limited medical resources. By 2024, Ecuador has reported over 30,000 deaths. These data from WHO reflect the widespread impact of COVID-19 globally and the differences in how various countries have managed the pandemic.

While the majority of patients experience mild (Upper respiratory tract infections, such as dry throat, sore throat, cough and fever) or moderate [Persistent high fever for more than 3 days, cough, shortness of breath, etc., but respiratory rate (RR) < 30 breaths/min and finger oxygen saturation > 93% on air intake at rest. Characteristic neocoronavirus-infected pneumonia manifestations are seen on imaging.] symptoms, in a subset of individuals, the condition progresses rapidly to severe illness with acute respiratory insufficiency, resulting in a mortality rate of 49%. Early detection and appropriate supportive treatment play a crucial role in reducing the incidence of severe cases and in-hospital mortality (7). Apart from its primary impact on the respiratory system, COVID-19 can also inflict damage on various other organs. COVID-19 may affect the nervous system through several pathways, including direct viral invasion, immune-mediated injury, vascular injury, or systemic inflammation of the central nervous system, resulting in neurologic symptoms such as headache, dizziness, confusion, and loss of smell or taste (8). COVID-19 may cause damage to the liver through direct infection of liver cells (via ACE2 receptor) or through systemic inflammation and immune response, manifested by elevated liver enzymes (e.g., ALT, AST) (9).

Several studies have reported the heightened vulnerability of individuals with pre-existing chronic conditions such as diabetes, hypertension, and cardiovascular disease to COVID-19 infection, with an elevated risk of developing critical illnesses or experiencing fatal outcomes (10–12).

By the end of 2022, the World Health Organization (WHO) had identified five “variants of concern”: Alpha (B.1.1.7), Beta (B.1.351), Gamma (P.1), Delta (B.1.617.2), and Omicron (B.1.1.529) (13). The Omicron variant that emerged in November 2021 rapidly progressed to becoming the predominant strain globally by early 2022, exhibiting significantly heightened transmissibility and immune evasion when compared to other “variants of concern.” (14) With the advent of Omicron, the rate of viral evolution has accelerated significantly, giving rise to many new Omicron subvariants. “Variants of Interest” (VOIs) have specific mutations in the genome that may affect viral transmissibility, immune escape ability, disease severity, or diagnostic, therapeutic, and vaccine efficacy, such as EG.5, BA.2.86, or KP.2. Currently, BA.2.86 and its progeny, JN.1, predominate and contain more than 30 additional mutations in the spiking protein compared to the BA.2 strain (15).

As per Chinese expert consensus (16), Based on the patient’s clinical presentation, respiratory status, organ function, and laboratory findings, COVID-19 is classified into mild, moderate, severe, and critical types (Critical types: one of the following conditions: 1. Respiratory failure and need for mechanical ventilation; 2. Shock; 3. Combined with other organ failure requiring ICU supervision and treatment). Despite a substantial decrease in the proportion of severe and critical cases relative to the initial stage of the 2019 coronavirus outbreak (17), COVID-19 persistently remains a critical and life-threatening disease.

There is limited research on the risk factors for severe outcomes in Omicron infections. Previous studies have identified various factors, including gender, age, comorbidities, pro-inflammatory cytokine interleukin-6 (IL-6), and D-dimer, as indicators for potential mortality risk factors in patients with severe COVID-19. The specific predictive significance of these indicators, particularly in severe Omicron infections, remains unclear. This merits further investigation, especially considering the constraints of limited medical resources and medical overcrowding.

In this study, our aim was to comprehensively investigate demographic factors, comorbidities, laboratory parameters, and clinical outcomes to identify risk factors associated with mortality. Unlike previous studies, we not only considered laboratory indicators upon patients’ initial hospitalization in our analysis but also included parameters related to the peak of the infection trajectory, which offer crucial predictive value for early identification of individuals at risk of severe illness. Consequently, we hope our results provide comprehensive insights into understanding the epidemiology and severity of COVID-19, offering a scientific basis for more effective prevention and control measures.

## 2 Materials and methods

### 2.1 Study design and participants

This case–control study involved patients hospitalized with severe COVID-19 between December 18, 2022, and January 31, 2023, at the affiliated hospitals in Tianjin, China. The diagnosis of severe COVID-19 was based on the guidelines provided by the WHO (18) and the “Treatment protocol for novel coronavirus pneumonia (trial 10th edition)” (16).

Inclusion criteria for the study required patients to meet the aforementioned diagnostic criteria. Exclusion criteria included pre-existing pneumonia prior to COVID-19 positivity, severe psychiatric illness, malignancy, pregnancy, and severe liver and kidney impairment before the onset of the disease.

**Figure 1:**
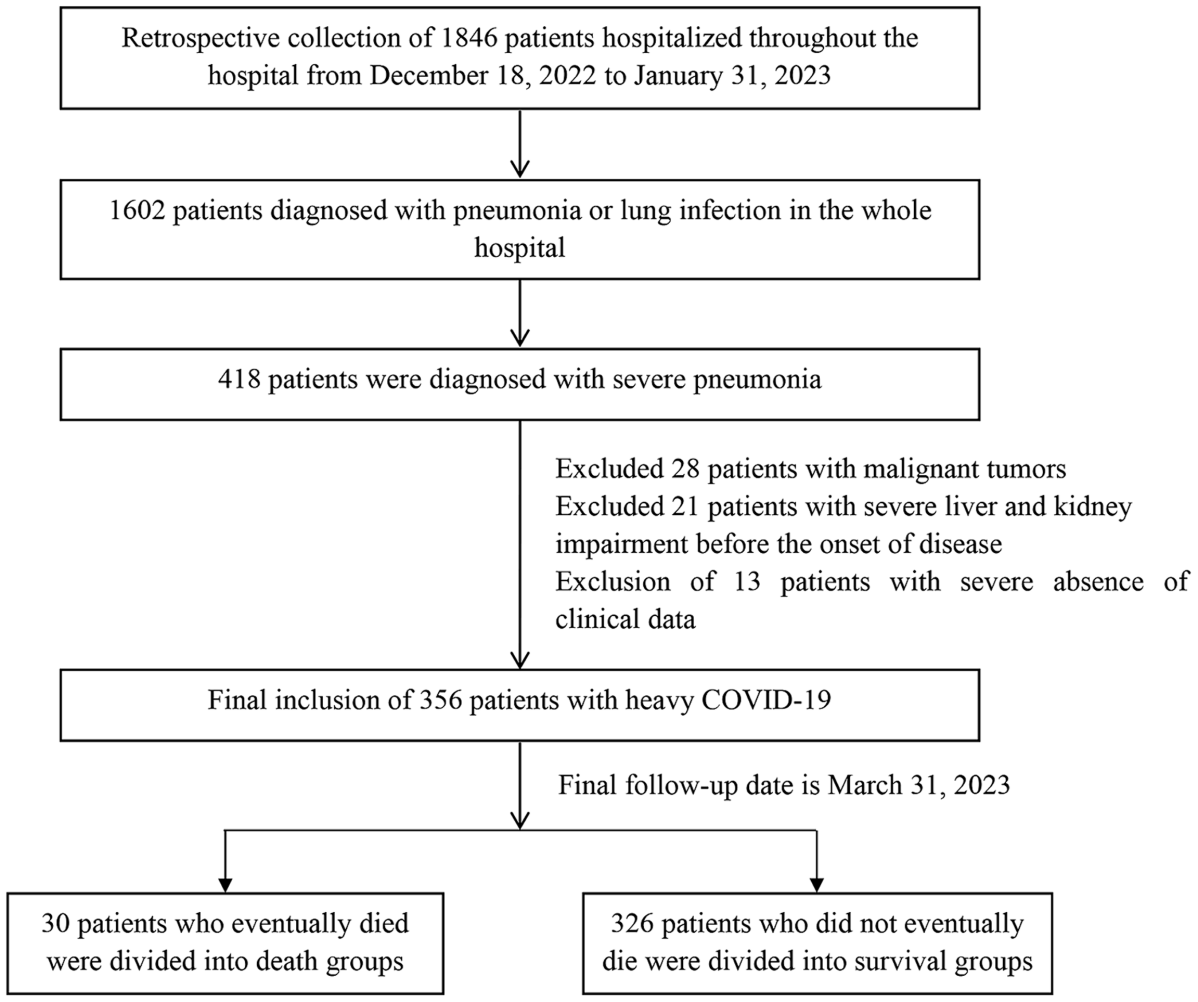
Flow chart of screening of the study population.

During the study period, a total of 1,846 patients were admitted to the hospital, with 1,602 patients diagnosed with pneumonia. Among them, 418 patients were identified as having severe COVID-19. We excluded 28 patients with malignancy, 21 patients with severe liver and kidney impairment prior to the disease onset, and 13 patients with significant clinical data deficiencies. Previously diagnosed by asking about disease history, laboratory and imaging tests. We excluded patients with malignant tumors and prior severe liver and kidney disease to reduce confounding factors and improve the efficiency and accuracy of the study. Thus, the final study sample consisted of 356 patients with severe infections. The screening flow chart of the study population is presented in Figure 1.

The data used in this study were compiled from multiple sources, including the Advantageous Diseases Health Care Research-based Database of the affiliated hospitals, the hospital information management system, and the electronic medical record databases of the institutions involved in the study. A team of three investigators was responsible for collecting, reviewing, and verifying all the original data. In cases where there were missing or inconsistent results, the original medical records were consulted for clarification. The accuracy of the information was verified with the treating physician and the patient’s family. Detailed checks were conducted to resolve any uncertainties or discrepancies in the records. The study was approved by the Ethics Committee of the affiliated hospitals in Tianjin (ethical approval number: TYLL2022[Z]011), and the requirement of informed consent from the study participants was waived.

### 2.2 Definitions

We referred to the “Diagnostic and treatment protocol for novel coronavirus pneumonia (Trial Version 10)” (16), and used the following diagnostic criteria for COVID-19: (1) presence of clinical manifestations associated with neo-coronavirus infection; and (2) presence of one or more of the following pathogenic and serological findings: (a) detection of neo-coronavirus nucleic acid; (b) positive neo-coronavirus antigen test result; (c) positive neo-coronavirus isolation and culture; or (d) a fourfold or higher increase in neo-coronavirus-specific IgG antibodies during the recovery period as compared to the acute period.

The diagnosis of severe COVID-19 was based on the guidelines provided by the WHO (18). The diagnostic criteria for severe COVID-19 that we used in this study were as follows: (1) presence of shortness of breath with a respiratory rate of ≥30 breaths/min. (2) finger oxygen saturation ≤ 93% at rest while breathing ambient air. (3) PaO2/inhaled oxygen concentration ≤ 300 mmHg (1 mmHg = 0.133 kPa). (4) progressive worsening of clinical symptoms and CT imaging of the lung showing significant progression of the lesion (> 50%) within 24 to 48 h.

We defined the duration of hospitalization as the period from admission of the patient into the facility until discharge or death. The duration of fever was defined as the number of days with fever from the onset of the patient’s perceived fever to the time of hospitalization.

### 2.3 Data collection

#### 2.3.1 Demographic indicators

Age, gender, ethnicity, and place of residence.

#### 2.3.2 Morbidity

Vital signs, duration of fever, and comorbidities such as respiratory insufficiency, heart failure, atrial fibrillation, hypertension, coronary artery disease, and diabetes.

#### 2.3.3 Laboratory indicators

Since patients were tested multiple times, we used data from the initial laboratory assessments at the time of admission and the most noteworthy results (either highest or lowest) recorded during hospitalization based on the significance of the corresponding indicators. These values were subscripted as F (first value), H (highest value), or L (lowest value) for the respective indicator. Details are given in Table 1.

**Table 1:** Statistical analysis of demographic indicators, morbidity, and laboratory indicators.

Indicators		N	total	N	dead	N	alive	*p* value	Test statistic value
Demographic features									
Age (years)		356	79.47 ± 7.99	30	82.53 ± 5.95	326	76.40 ± 8.66	0.002*	3.198^a^
Sex		356		30		326			
Male			237 (66.57%)		20 (66.67%)		217 (66.56%)	1	0.000^b^
Female			119 (33.43%)		10 (33.33%)		109 (33.44%)	1	0.000^b^
Days of hospitalization (days)		356	14.0 (14)	30	7.0 (11.0)	326	18.5 (11.75)	<0.001*	−4.506
Symptoms									
Fever time (days)		270	7.0 (5.0)	20	4.0 (5.0)	250	7.0 (5.0)	0.015*	−2.444
**Comorbidities: (Yes/No)**		291							
Respiratory insufficiency			104 (29.21%)	30	28 (93.33%)	326	76 (23.31%)	<0.001*	30.240^b^
Heart failure			45 (12.64%)	30	12 (40.00%)	326	33 (10.12%)	0.007*	7.200^b^
Coronary artery disease			149 (41.85%)	30	19 (63.33%)	326	130 (39.88%)	0.071	3.270^b^
Atrial fibrillation			52 (14.61%)	30	9 (30.00%)	326	43 (13.19%)	0.117	2.455^b^
Hypertension			149 (41.85%)	30	19 (63.33%)	326	130 (39.88%)	0.071	3.270^b^
Diabetes mellitus			197 (55.34%)	30	12 (40.00%)	326	185 (56.75%)	0.196	1.669^b^
Laboratory examinations									
PCO2, mmHg	PCO2_F_	331	38.0 (13.2)	27	34.8 (16.7)	304	39.3 (9.35)	0.101	−1.642
	PCO2_H_	331	40.9 (13.0)	27	38.2 (26.6)	304	41.5 (10.17)	0.266	−1.111
PAO2, mmHg	PAO2_F_	331	68.0 (36.3)	27	56.1 (29.0)	304	83.35 (33.28)	0.001*	−3.376
	PAO2_L_	331	57.0 (35.1)	27	47.7 (22.2)	304	64.45 (29.4)	0.002*	−3.124
SF, μg/L	SF_F_	285	475.65 (760.54)	24	484.87 (1260.42)	261	439.4 (437.3)	0.421	−0.805
	SF_H_	285	488.28 (793.20)	24	599.93 (1260.42)	261	467.40 (437.3)	0.312	−1.011
WBC, ×10^9/L	WBC_F_	356	7.57 (5.94)	30	8.4 (6.72)	326	7.43 (4.9)	0.069	−1.819
	WBC_H_	356	11.5 (7.21)	30	14.26 (13.9)	326	10.89 (4.59)	0.019*	−2.336
ALB, g/L	ALB_F_	356	30.19 ± 4.69	30	30.14 ± 4.81	326	30.24 ± 4.64	0.935	−0.082^a^
	ALB_L_	356	27.35 ± 4.01	30	26.67 ± 3.49	326	28.03 ± 4.42	0.191	−1.323^a^
CK, U/L	CK_F_	356	101.5 (180.5)	30	109.20 (197.23)	326	71.05 (184.47)	0.267	−1.109
	CK_H_	356	169.7 (360.0)	30	363.50 (724.53)	326	75.1 (212.27)	0.001*	−3.364
GFR, ml/min	GFR_F_	355	72.70 ± 27.43	29	65.08 ± 28.03	326	80.06 ± 25.14	0.035*	−2.163^a^
	GFR_L_	355	66.27 ± 31.42	29	57.91 ± 32.64	326	74.35 ± 28.44	0.043*	−2.065^a^
CRP, mg/L	CRP_F_	309	42.63 (98.37)	16	90.30 (134.63)	293	27.35 (65)	0.027*	−2.218
	CRP_H_	309	53.11 (133)	16	106.82 (144.21)	293	41.67 (80.97)	0.021*	−2.316
hs-CRP, mg/L	hs-CRP_F_	309	10 (0)	16	10 (0.73)	293	10 (0)	0.919	−0.102
	hs-CRP_H_	309	10 (0)	16	10 (0)	293	10 (0)	0.970	−0.037
PCT, ng/mL	PCT_F_	307	0.08 (0.5)	24	0.18 (1.51)	283	0.05 (0.09)	0.006*	−2.770
	PCT_H_	307	0.13 (1.23)	24	0.81 (2.49)	283	0.08 (0.29)	0.008*	−2.650
BNP, pg./mL	BNP_F_	302	184.70 (699.6)	30	347.25 (1839.83)	272	93.9 (299.1)	0.012*	−2.510
	BNP_H_	302	311.0 (1197.9)	30	616.15 (2328.15)	272	236.6 (422)	0.023*	−2.274
D-dimer, mg/L	D-dimer_F_	356	1.63 (3.0)	30	2.29 (3.94)	326	1.31 (1.44)	0.040*	−2.055
	D-dimer_H_	356	2.99 (4)	30	4.17 (6.05)	326	1.94 (2.69)	0.016*	−2.403

*p*-values marked with * indicate values less than 0.05. The rest represents the z-value.

a represents the *t*-value.

b represents the Pearson χ^2^ value.

#### 2.3.4 Clinical outcomes

We categorized the clinical outcomes as survival and death. The outcome events of patients who survived or died at discharge were recorded.

### 2.4 Statistical analysis

Continuous variables were represented as the median and interquartile range (IQR) for skewed distributed data or the mean ± standard deviation (SD) for normally distributed data. Categorical variables were represented as numbers and percentages (%). Means for continuous variables were compared using independent sample t tests. Medians for continuous variables were compared using the non-parametric Mann–Whitney U test. Categorical variables were compared using χ2.

Univariable and multivariable logistic regression model analyses were conducted to assess the odds ratio (OR) and 95% confidence interval (CI). These analyses aimed to investigate the risk factors associated with morbidity and mortality in patients with severe COVID-19.

We used receiver operating characteristic (ROC) curves to evaluate the predictive value of age, coexisting respiratory insufficiency, WBC, CK, and PAO2 indicators for predicting death occurrence in patients with severe COVID-19. The AUC was compared using the Delong test. Statistical significance was defined as a *p* value <0.05.

## 3 Results

### 3.1 Demographic indicators

As shown in Table 1, we included a total of 356 patients with severe COVID-19, with 30 cases in the deceased group and 326 cases in the survival group, resulting in a mortality rate of 8.4%. Among the 356 patients, there were 237 males (66.57%) and 119 females (33.43%), signifying a predominance of males, with an incidence rate in males that was approximately twice that of females.

The age of the study subjects ranged from 57 to 94 years, with a mean age of 79.47 ± 7.99 years. The mean age in the deceased group was 82.53 ± 5.95 years, while in the survival group, it was 76.40 ± 8.66 years. We found a statistically significant association between age and clinical outcome (*p* = 0.002, *t* = 3.198), indicating that older patients with severe COVID-19 had a higher risk of death.

### 3.2 Comorbidity

Among patients with severe COVID-19, 81.74% had at least one underlying disease. Diabetes mellitus was the most prevalent, while hypertension and coronary artery disease had equal prevalence. In the deceased group, 19 cases (63.33%) had coronary artery disease, 12 cases (40.00%) had diabetes mellitus, 19 cases (63.33%) had hypertension, and 9 cases (30.00%) had atrial fibrillation. In the survival group, 130 cases (39.88%) had coronary artery disease, 185 cases (56.75%) had diabetes mellitus, 130 cases (39.88%) had hypertension, and 43 cases (13.19%) had atrial fibrillation. However, the differences in the prevalence of these four diseases between the two groups were not statistically significant (*p* > 0.05).

In the deceased group, 28 cases (93.33%) had respiratory insufficiency, and 12 cases (40.00%) had heart failure. In the survival group, 76 cases (23.31%) had respiratory insufficiency, and 33 cases (10.12%) had heart failure. We found statistically significant differences in the prevalence of respiratory insufficiency and heart failure between the two groups (*p* < 0.001 and *p* = 0.007, respectively).

### 3.3 Laboratory indicators

Among the blood gas indicators, the deceased and survival groups differed significantly with respect to PAO2_F_ (*p* = 0.001, *Z* = −3.376) and PAO2_L_ (*p* = 0.002, *Z* = −3.124). However, the arterial partial pressure of carbon dioxide (PCO2_F_) and PCO2_H_ did not exhibit statistical significance.

Among inflammatory markers, differences in WBC_H_ (*p* = 0.019, *Z* = −2.336), C-reactive protein (CRP_F_; *p* = 0.027, *Z* = −2.218), CRP_H_ (*p* = 0.021, *Z* = −2.316), procalcitonin (PCT_F_; *p* = 0.006, *Z* = −2.770), and PCT_H_ (*p* = 0.008, *Z* = −2.650) were found to be statistically significant, while differences in WBC_F_, high-sensitivity C-reactive protein (hs-CRP_F_), and hs-CRP_H_ were not statistically significant.

Differences between the groups with respect to renal function indicators, namely, glomerular filtration rate (GFR_F_; *p* = 0.035, *t* = −2.163) and GFR_L_ (*p* = 0.043, *t* = −2.065), as well as cardiac function indicators, specifically, B-type natriuretic peptide (BNP_F_; *p* = 0.012, *Z* = −2.510) and BNP_H_ (*p* = 0.023, *Z* = −2.274), were statistically significant. These results suggest that cardiac and renal insufficiency were associated with mortality.

The levels of coagulation markers D-dimer_F_ (*p* = 0.040, *Z* = −2.055) and D-dimer_H_ (*p* < 0.001, *Z* = −4.022) differed significantly between the groups (*p* < 0.05), with higher levels observed in the deceased group as compared to the survival group. Differences in CK_H_ (*p* = 0.016, *Z* = −2.403) exhibited statistical significance, while those in CK_F_ levels did not. This suggests that skeletal muscle damage occurred in the later stages of infection and was associated with mortality. Differences in levels of serum ferritin (SF_F_), SF_H_, albumin (ALB_F_), and ALB_L_ were not statistically significant.

### 3.4 Multivariate logistic regression analysis

Based on the obtained results, we incorporated variables with a significance level of *p* < 0.1 in a multifactorial logistic regression analysis, as shown in Table 2. The differences in age of patients were statistically significant in the comparison between the two groups (*p* = 0.018, OR: 12.336, 95% CI: 1.528–99.571), indicating that patients with severe COVID-19 had an increased risk of death as age increased. For every 10-year increase in age, the risk of death increased 12.336-fold.

**Table 2:** Univariable and multivariable logistic regression analysis.

Indicators	Univariate logistic regression			Multivariate logistic regression		
	OR	95% CI	*p* value	OR	95% CI	*p* value
Age (years)	1.123	1.035–1.218	0.005	12.336	1.528–99.571	0.018
Respiratory insufficiency (Yes/No)	46.000	8.699–243.250	<0.001	241.32	7.101–8201.031	0.002
PAO2_F_, mmHg	0.968	0.944–0.993	0.012	9.842	1.422–68.121	0.021
WBC_H_, ×10^9/L	1.142	1.030–1.266	0.012	9.811	1.034–93.087	0.047
CK_H_,U/L	1.002	1.000–1.004	0.026	14.301	1.310–156.118	0.029

OR refers to the ratio, and CI represents the confidence interval. The statistical analysis was adjusted for age, sex, and comorbidities, including respiratory failure, heart failure, coronary artery disease, atrial fibrillation, hypertension, and diabetes mellitus, as well as laboratory test indices. The adjustments were made to ensure accurate and comprehensive analysis of the data.

The presence of respiratory insufficiency complications also showed a statistically significant difference between the two groups (*p* = 0.002, OR: 241.32, 95% CI: 7.101–8201.031), with a higher risk of death and poorer prognosis observed in infected individuals with respiratory insufficiency.

When comparing the laboratory test results at the time of hospitalization, there was a statistically significant difference in PAO2_F_ levels between the deceased and survival groups (*p* = 0.021, OR: 9.842, 95% CI: 1.422–68.121). PAO2 was lower in the deceased group compared to the survival group, indicating that lower PAO2 levels were associated with an increased likelihood of adverse outcomes. Differences in WBC_H_ were also significant between the two groups (*p* = 0.047, OR: 9.811, 95% CI: 1.034–93.087), with higher white blood cell counts observed in the deceased group. WBC was significantly associated with poor clinical outcomes.

We found that CK levels were higher in the deceased group compared to the survival group, with a statistically significant difference between the two groups, and CK_H_ significantly influenced prognostic outcomes (*p* = 0.029, OR: 14.301, 95% CI: 1.310–156.118).

Overall, logistic univariate and multifactorial regression analyses demonstrated that age, coexisting respiratory insufficiency, PAO2, WBC, and CK levels were significantly associated with poor clinical outcomes.

### 3.5 ROC curve analysis

**Figure 2:**
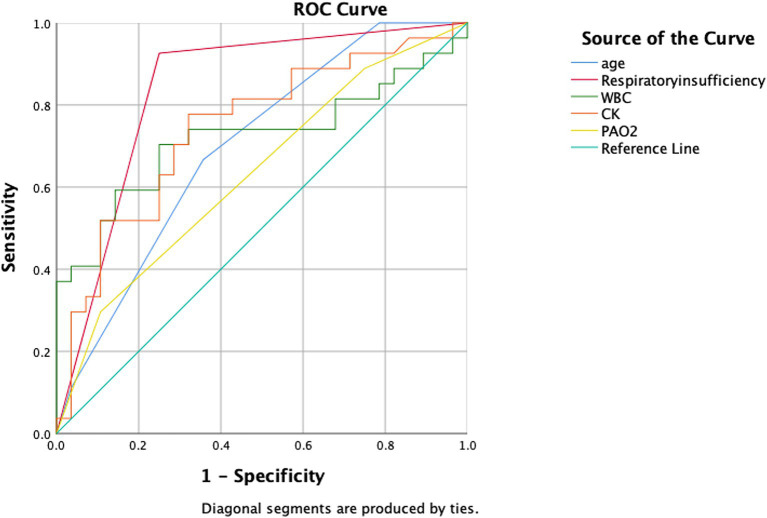
ROC curves comparing the five aforementioned indicators as predictors of death in patients with hospitalized severe COVID-19. The abbreviations used are as follows: CK, creatine kinase; PAO2, arterial oxygen partial pressure; and WBC, white blood cell count.

Based on our multifactorial logistic regression analysis, we included the resulting five indicators in the ROC curve analysis. Figure 2 and Table 3 present the AUC and their corresponding 95% confidence intervals for age, coexisting respiratory insufficiency, WBC_H_, CK_H_, and PAO2_F_ as predictors of poor prognosis in patients hospitalized with severe COVID-19. The AUCs for age, respiratory insufficiency, WBC_H_, CK_H_, and PAO2_F_ were 0.698 (95% CI 0.560–0.837), 0.838 (95% CI 0.725–0.951), 0.721 (95% CI 0.578–0.864), 0.744 (95% CI 0.611–0.877), and 0.633 (95% CI 0.486–0.780), respectively.

**Table 3:** AUC, *p* value, 95% CI and cutoff point for selected indicators.

	AUC	*p* value	95% CI	Cutoff point	Sensitivity	Specificity
Age (years)	0.698	0.012	0.560–0.837	81.5	0.6	0.733
Respiratory insufficiency (0/1)	0.838	<0.001	0.725–0.951	1^*^	0.923	0.75
WBC_H_ (×10^9^/L)	0.721	0.005	0.578–0.864	12.53	0.633	0.767
CK_H_ (U/L)	0.744	0.002	0.611–0.877	299.35	0.567	0.9
PAO2_F_ (mmHg)	0.633	0.091	0.486–0.780	50.35	0.423	0.107

The asterisk (*) represents the inclusion of combined respiratory insufficiency in the categorical variables.

Among these indicators, respiratory insufficiency had the highest predictive value, while WBC_H_ and CK_H_ exhibited high predictive value. Age and PAO2_F_ showed a lower predictive value for clinical outcomes in patients hospitalized with severe COVID-19. The optimal cut-off value for age was determined to be 81.5 years, with a sensitivity of 0.6 and a specificity of 0.733. For WBC_H_, the optimal cut-off value was 12.53 × 10^9^/L, with a sensitivity of 0.633 and a specificity of 0.767. CK_H_ had an optimal cut-off value of 299.35 U/L, with a sensitivity of 0.567 and a specificity of 0.9. The optimal cut-off value for PAO2_F_ was determined to be 50.35 mmHg, with a sensitivity of 0.423 and a specificity of 0.107.

In summary, coexisting respiratory insufficiency as an underlying disease and laboratory test results for WBC_H_ and CK_H_ demonstrated high predictive value, while age and PAO2_F_ exhibited moderate predictive value for clinical outcomes in patients hospitalized with severe COVID-19.

## 4 Discussion

The overall case-fatality rate has been reported as 2.3% in China (7), and the mortality rate in Italy is 7.2% (19), and 49.0–61.5% in critically ill cases (20). In this study, we observed an in-hospital mortality rate of 8.4% among patients with severe COVID-19, higher than the overall mortality rate reported for China but significantly lower than the mortality rate reported for critical cases. Early and aggressive treatment during the severe stage of COVID-19 can significantly reduce overall mortality.

Our study also highlights the likelihood of infection and mortality from severe COVID-19 in males as being approximately twice that of females. This observation is consistent with previous research (21). The significant impact of gender on clinical outcomes may be attributed to the higher prevalence of comorbidities in men compared to women (22).

We also found that the risk of death in patients with COVID-19 increased by approximately 12-fold with every 10-year increase in age. This finding is consistent with the results of previous research (23). One study (24) showed that older patients tend to exhibit subpleural lesions and are more likely to experience higher disease severity when compared to younger patients. This may be related to the following reasons: (1) The prevalence of underlying diseases such as hypertension, diabetes, and coronary heart disease increases with age (25). (2) Baseline levels of pro-inflammatory cytokines tend to be elevated in the older adult, leading to a delayed immune response to pathogenic threats or tissue damage (26). (3) Older adult patients exhibit lower immunogenicity and reduced efficacy of novel coronavirus vaccines compared to other age groups (27).

These findings of our study shed light on the impact of gender and age on the increased risk of death among patients with COVID-19, providing valuable insights for early prediction of adverse outcomes.

One study (20) has shown that patients with underlying diseases like hypertension, cardiovascular disease, and diabetes have a significantly higher risk of developing severe COVID-19 and increased mortality rates compared to those without underlying diseases. However, we did not observe a significant difference between the two groups with respect to comorbid conditions. The similar proportions of hypertension and coronary heart disease in both groups may be attributed to the high prevalence of these underlying conditions in the overall population, resulting in no statistical difference.

However, this study revealed a contradictory finding: patients with comorbid diabetes had a lower mortality rate compared to non-diabetic patients, which is inconsistent with previous research (28). One possible explanation for this discrepancy could be the use of metformin, which has been reported to inhibit the replication of the SARS-CoV-2 coronavirus (29). The use of metformin in patients with early COVID-19 may mitigate the risk of severe illness (30).

Our data demonstrated that the concentration of PAO2_F_ was lower in patients in the deceased group compared to the survival group. This finding aligns with a recent study (31) and suggests impaired pulmonary function in fatal cases. Reduced levels of arterial oxygen partial pressure, as evidenced in blood gas analysis, can lead to electrolyte imbalances and hypoxia, thereby increasing the risk of death in patients with severe COVID-19. Another study reported that higher mortality rates were linked to more severe lung injuries upon admission and lower oxygen saturation levels (32).

In our study, we identified a significant association between respiratory insufficiency and adverse clinical outcomes, which is in line with previous research (33). A subset of patients with severe COVID-19 develops life-threatening respiratory insufficiency, acute respiratory distress syndrome (ARDS), and multi-organ failure (34). Analysis of chest CT scans indicated that individuals who died had more extensive lung involvement, providing evidence for the development of severe disease in these cases (35). Autopsy studies of patients who succumbed to a severe SARS-CoV-2 infection revealed diffuse alveolar injury and a high burden of thrombosis in the pulmonary capillaries (36).

Our analysis revealed elevated levels of WBC, CRP, and PCT in individuals who succumbed to the disease compared to those who survived, consistent with prior research (37). It has been found that patients with severe COVID-19 exhibited higher levels of inflammatory cytokines when compared to individuals with mild to moderate COVID-19, suggesting the involvement of a “cytokine storm” as a potential causal factor (38). Cytokine release syndrome has also been implicated in the pathology of COVID-19 (39). In cases where COVID-19-induced lung injury progresses and necessitates invasive mechanical ventilation, anti-inflammatory treatment to suppress the cytokine storm is recommended to mitigate disease progression toward ARDS and multi-organ failure (40).

However, it is important to note that in this study, we found that the white blood cell count at admission had limited predictive value, suggesting that WBC is less sensitive than CRP and PCT in detecting early-stage inflammation. Additionally, hs-CRP also did not exhibit predictive value, and this may be likely due to the fact that CRP levels were already elevated in all severe infections, surpassing the upper limit of hs-CRP detection.

We observed statistically significant differences between the two study groups in the laboratory index CK_H_. Increased CK_H_ levels were associated with a higher risk of death in patients, suggesting that CK_H_ can serve as a prognostic indicator for patients with severe COVID-19. The author conducted an extensive literature search and found only a few studies reporting CK as a predictor. Results of logistic univariate regression analysis in a previous study (41) identified CK as an indicator of disease severity, and elevated CK levels at admission were associated with poor outcomes. However, after adjusting for multifactorial analysis, the statistical differences were no longer significant. Some studies have indicated that CK levels decrease with age (42). Nevertheless, findings from another study highlighted that CK levels increase with age in patients with COVID-19, potentially due to decreased ability to cope with stressors and persistent elevation of pro-inflammatory factors (43).

There are several possible reasons to explain why CK is a risk factor affecting the prognosis of patients with severe COVID-19: (1) Cardiac injury is a common complication in patients with COVID-19 and is associated with an increased risk of disease severity. Researchers have estimated that approximately 23% of confirmed COVID-19 patients exhibit cardiac injury, with 13% displaying elevated CK levels (20, 44). (2) In patients with severe COVID-19, the intensified anti-inflammatory process necessitates increased energy consumption by body tissues, leading to elevated CK levels in the blood (45). (3) Skeletal muscle damage and rhabdomyolysis are observed in patients with severe COVID-19, resulting in the release of intracellular enzymes, such as serum CK, into the bloodstream. Consequently, CK levels are significantly increased, leading to systemic complications, including rhabdomyolysis (RM) (46).

Our results indicate that a reduced glomerular filtration rate is associated with a poor clinical outcome in patients with severe COVID-19, and this is consistent with previous findings (47). The glomerular filtration rate serves as a measure of the kidneys’ efficiency in filtering water, toxins, and metabolites, thus providing valuable insights into kidney function and early detection of kidney disease. Several studies have demonstrated that underlying chronic kidney disease is an independent risk factor for mortality, and severe COVID-19 can negatively impact renal function, leading to a significant decline in glomerular filtration rate (48–50). Moreover, conditions associated with reduced glomerular filtration rate, such as glomerulonephritis (51) and end-stage renal disease (52), are more prevalent in patients who do not survive compared to those who do.

Notably, among patients who died, it was found that a higher proportion had a history of using renin-angiotensin-aldosterone system inhibitors. Angiotensin converting enzyme (ACE) 2 receptors, which serve as a major pathway for novel coronavirus invasion, are upregulated in patients with severe COVID-19 due to the higher prevalence of comorbidities such as hypertension, diabetes, and cardiovascular disease. The increased use of ACE inhibitors or angiotensin II receptor blockers in these patients may further enhance the invasion of the novel coronavirus, potentially exacerbating the progression of the disease (53–55).

The results of our study revealed that there was no significant difference between the deceased and survival groups in terms of D-dimer, a crucial parameter. This lack of statistical difference can be attributed to two factors: First, patients with severe COVID-19 tend to exhibit higher levels of inflammation, leading to a substantial increase in D-dimer. Since our study population consisted of patients with severe COVID-19, it is likely that the increased inflammation contributed to the significant elevation of D-dimer across the entire sample. Second, in response to the notable increase in D-dimer levels, the treating physicians administered anticoagulant drugs to reduce these levels as much as possible. As a result, D-dimer values decreased or even returned to normal, thereby eliminating abnormally high values from the dataset.

Although we did not find a predictive value for D-dimer in this study, it is important to acknowledge that elevated D-dimer levels are significantly associated with adverse clinical outcomes in COVID-19 (56). Nevertheless, further investigation is warranted to explore the relationship between anticoagulation therapy, decreased mortality, and D-dimer levels.

## 5 Limitations and future prospects

The present study has several limitations that should be acknowledged. Firstly, the study population was derived from affiliated hospitals in Tianjin, and this potentially constrains the generalizability of the findings to patients of different races or regions. Moreover, the sample size of this study is relatively small, and the findings may be biased in a way that makes it difficult to fully represent a wider range of situations. Secondly, retrospective studies are susceptible to confounding biases, including factors such as age, gender, and comorbidities. Although extensive adjustments were made to account for potential confounders in our analysis, there may still be unmeasured confounding variables. Thirdly, due to the nature of retrospective studies, not all patients underwent comprehensive laboratory testing due to medical requirements and various complicating factors. Fourthly, all patients included in this study were confirmed positive through antigen testing, and nucleic acid sampling was not performed. As a result, some patients may have been misclassified as infected with SARS-CoV-2 due to false-positive antigen test results, while others may have been missed due to false-negative results.

Moreover, we could not undertake a long-term follow-up of patient survival prognosis as the study’s endpoint was in close proximity to the analysis. Future research incorporating long-term follow-up can help uncover potential prognostic indicators. Additionally, it is important to note that we conducted this study at a single center, underscoring the necessity for further confirmation of the identified independent risk factors in other clinical trials. Therefore, large-scale prospective cohort studies and randomized controlled trials are warranted to gain a better understanding of the independent risk factors associated with mortality in patients with severe COVID-19 infections.

## 6 Conclusion

In conclusion, for patients with severe COVID-19, we identified age, respiratory insufficiency, oxygen partial pressure, white blood cell count, and creatine kinase as significant risk factors for a poor prognosis. Combining these factors with other investigations, such as chest CT, can effectively predict mortality in patients with severe COVID-19 pneumonia.

## Data Availability

The original contributions presented in the study are included in the article/supplementary material, further inquiries can be directed to the corresponding author.

## Glossary

COVID-19: coronavirus disease 2019
WBC: White blood cell count
CK: Creatine kinase
PAO2: Arterial Oxygen Partial Pressure
AUC: area under curve
SARS-CoV-2: severe acute respiratory syndrome coronavirus type 2
WHO: World Health Organization
IL-6: Interleukin-6
IQR: Interquartile Range
SD: Standard Deviation
OR: Odds Ratio
CI: Confidence Interval
ROC: Receiver Operating Characteristic
PCO2: Arterial Partial Pressure of Carbon Dioxide
SF: Serum ferritin
ALB: Albumin
GFR: Glomerular filtration rate
CRP: C-reactive protein
hs-CRP: High-sensitivity C-reactive protein
PCT: Procalcitonin
BNP: B-type natriuretic peptide
D-dimer: D-dimer
ARDS: Acute Respiratory Distress Syndrome
ACE: Angiotensin converting enzyme
